# High intake of dietary fibre from fruit and vegetables reduces the risk of hospitalisation for diverticular disease

**DOI:** 10.1007/s00394-018-1792-0

**Published:** 2018-08-06

**Authors:** Mahmood W. Mahmood, Mirna Abraham-Nordling, Niclas Håkansson, Alicja Wolk, Fredrik Hjern

**Affiliations:** 1 Department of Clinical Sciences, Division of Surgery, Danderyd Hospital, Karolinska Institutet, Stockholm, Sweden; 2 Division of Coloproctology, Center of Digestive Diseases, Karolinska University Hospital, Stockholm, Sweden; 3 Department of Molecular Medicine and Surgery, Karolinska Institutet, Stockholm, Sweden; 4 The National Institute of Environmental Medicine (IMM), Karolinska Institutet, Stockholm, Sweden; 5 Department of Surgery and Urology, Danderyd University Hospital, 182 88 Stockholm, Sweden

**Keywords:** Diverticular disease, Diverticulitis, Risk factors, Dietary fibres

## Abstract

**Backgrounds and aims:**

High intake of dietary fibres has been associated with a reduced risk of DD. However, reports on which type of dietary fibre intake that is most beneficial have been conflicting. The aim of this study was to investigate the association between different dietary fibres and hospitalisation due to diverticular disease (DD) of the colon.

**Methods:**

This was a major cohort study. The Swedish Mammography Cohort and the Cohort of Swedish Men were linked to the Swedish Inpatient Register and the Causes of Death Register. Data on the intake of dietary fibre were collected through questionnaires. The effect of intake (in quartiles) of different types of dietary fibre on the incidence of hospitalisation due to DD was investigated using multivariable Cox regression. Estimates were adjusted according to age, BMI, physical activity, co-morbidity, intake of corticosteroids, smoking, alcohol intake and education level.

**Results:**

Women with intake of fruit and vegetable fibres in the highest quartile (median 12.6 g/day) had a 30% decreased risk of hospitalisation compared to those with the lowest intake (4.1 g/day). Men within the highest quartile (10.3 g/day) had a 32% decreased risk compared to those with a low intake (2.9 g/day). High intake of fibres from cereals did not affect the risk.

**Conclusion:**

A high intake of fruits and vegetables may reduce the risk of hospitalisation due to DD. Intake of cereals did not influence the risk.

## Introduction

Diverticular disease (DD) of the colon is common among adults. The lifetime risk to develop complications such as diverticulitis is up to 4% [1]. Health-care expenditures for this disorder were estimated to be $2.7 billion in 2009 in the USA only [2].

Dietary and lifestyle factors affect the risk of DD, though recent studies have found a hereditary component [3–5]. It is stated that low dietary intake of fibre increases the risk of DD, in terms of the development of diverticula in predominately the sigmoid colon as well as the promotion to diverticulitis and its complications [6–8]. However, recent studies have not seen this association [9]. Moreover, high intake of dietary fibre is thought to decrease the risk of recurrence after medically managed diverticulitis [10].

Other studies have found other risk factors for symptomatic DD in the diet, such as high intake of unprocessed red meat [11]. Non-dietary risk factors include smoking, steroid intake, low physical activity and obesity [12–14].

The low intake of dietary fibre results in small stools requiring high pressure and cause the mucosa to herniate through the weak areas in the bowel wall [8, 15]. Moreover, Aldoori et al. found that dietary fibre could affect the risk of the disease [7, 16]. In a recent study, a high fibre intake reduced the risk of DD, the reduced risk being strongest for cereal and fruit fibre, compared to vegetable and potato fibre [17].

The aim of the present study was to determine if different fibre types affect the risk of hospitalisation due to DD in two major Swedish cohorts of middle-aged and elderly men and women.

## Methods

### Cohorts

This is a prospective cohort study based on two major cohorts: the Swedish Mammography Cohort (SMC), and the Cohort of Swedish Men (COSM).

### The Swedish Mammography Cohort (SMC)

**Fig. 1:**
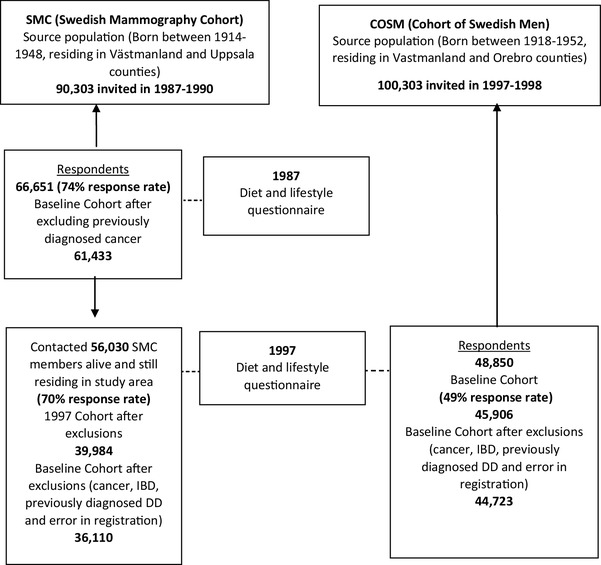
Description of recruitment and data collection for the Swedish Mammography Cohort (SMC 1987–1990) and the Cohort of Swedish Men (COSM 1997–1998)

SMC is a major prospective cohort consisting of 66,651 women in central Sweden born between 1914 and 1948. The cohort was established in 1987–1990. Women were invited by mail to participate in a mammography screening programme. The invitation included a questionnaire regarding diet and alcohol intake, parity, weight, height, education level and marital status. After excluding women with a previous diagnosis of cancer, the SMC included 61,433 women. In 1997, a second questionnaire was sent to those women still alive and living within the study area. Information about diet and alcohol intake, physical activity, medical history, height, weight, education level and lifestyle factors such as cigarette smoking history and use of some medications and dietary supplements was updated and completed. In the present study, the 1997 questionnaire was used as baseline (Table 1). In all, 39,984 of 56,030 women (71.4%) living in the study area responded to the follow-up questionnaire in 1997; 219 women were too sick to fill in the questionnaire and 548 declined to answer (Fig. 1) [18]. Women with IBD, cancer, previously diagnosed DD (*N* = 478) or error in the registration were excluded, leaving a final cohort of 36,110 women.

**Table 1:** Baseline characteristics by intake of fibres, Swedish Mammography Cohort (SMC), 1997

Dietary fibre intake, quartiles	Q1	Q2	Q3	Q4
	(*n* = 9028)	(*n* = 9027)	(*n* = 9027)	(*n* = 9028)
Total dietary fibre (median, g)	16.15	19.94	23.1	28.29
Total person-years	71.461	72.010	72.142	72.176
Mean person-years	7.92	7.98	7.99	7.99
Mean age (years), range 49–83	60.9	61.6	62.6	62.2
Smoking status, % current smokers	29.2	23.0	19.9	18.9
BMI	24.9	25.0	25.1	25.1
Diabetes (%)	2.4	3.2	3.9	6.5
Hypertension (%)	19.9	20.5	21.3	22.3
Use of steroids (%)	14.0	13.5	13.7	13.7
Mean physical activity (min/day)	49.5	54.9	58.5	65.3
Mean alcohol consumption (g/day)	7.4	6.3	5.5	4.7
University-level education (%)	17.6	18.9	18.8	18.6

### The Cohort of Swedish Men (COSM)

COSM is a prospective cohort consisting of 48,850 Swedish men born in 1918–1952 and was established in 1997–1998. Men living in central Sweden answered a questionnaire regarding diet, smoking, alcohol intake, physical activity, dietary supplements, some medications, height, weight and education level. The response rate was 49%.

After excluding those with an incorrect or missing national registration number, cancer diagnosis (except non-melanoma skin cancer) before baseline or other missing data, the final cohort consisted of 45,906 men. Men with IBD, cancer or previously diagnosed DD (*N* = 334) and error in the registration were excluded, leaving a final cohort of 44,723 men.

The baseline characteristics of the two cohorts are presented in Tables 1 and 2.

**Table 2:** Baseline characteristics by intake of fibres, Cohort of Swedish Men (COSM), 1997

Dietary fibre intake, quartiles	Q1	Q2	Q3	Q4
	(*n* = 11,180)	(*n* = 11,181)	(*n* = 11,181)	(*n* = 11,181)
Total dietary fibre (median, g)	21.51	27.54	32.38	39.63
Total person-years	83.593	84.241	85.063	85.021
Mean person–years	7.48	7.53	7.61	7.60
Mean age (years), range 45–79	59.9	60.4	60.5	60.3
Smoking status, % current smokers	31.7	24.9	21.6	19.9
BMI	25.9	25.7	25.7	25.7
Diabetes (%)	7.5	7.4	8.9	12.7
Hypertension (%)	23.9	24.3	24.0	26.1
Use of steroids (%)	8.2	7.8	7.2	7.3
Mean physical activity (min/day)	49.7	55.3	57.9	63.6
Mean alcohol consumption (g/day)	19.1	15.5	13.9	11.6
University-level education (%)	14.7	16.8	17.2	16.3

### Assessment of dietary fibre intake

Fibre consumption was assessed with a food frequency questionnaire. Participants in the two cohorts indicated their average consumption of 96 foods and beverages over the previous year. Participants could choose from eight predefined frequency categories ranging from never to three or more times per day. The amount of intake of fibres from cereals and fruits and vegetables, respectively, was estimated in grams per day for each patient. The patients were divided into four quartiles (Q1–Q4). Q1 contained patients who ate the lowest amount of fibres and Q4 contained patients who ate a larger amount of fibres. The amount of every quartile of fibre intake from cereals and fruit/vegetables was analysed separately. In a validation study in a subsample of 129 women from the SMC using a similar FFQ (including 60 foods), the Pearson correlation coefficients between the FFQ and four 1-week diet records (completed 3–4 months apart) ranged from 0.4 to 0.5 for fruit items, from 0.4 to 0.6 for vegetable items, 0.5 for hard whole grain rye bread, 0.5 for soft whole grain bread, 0.6 for porridge and 0.7 for cold breakfast cereals (Wolk unpublished data).

The validity of FFQs was assessed for foods, nutrients, dietary supplements, glycaemic index and glycaemic load by comparison with multiple 24-h recall interviews and/or diet records [19–23].

### Follow-up and ascertainment of hospital admissions

Cohorts were linked to the National Patient Registry (NPR) and the Causes of Death Registry (CDR). The NPR was established in 1964, but became nationwide since 1987 and contains information of all hospital visits in Sweden. The register covers more than 99% of all hospital discharges in Sweden and has been shown to be valid for most diagnoses [24]. CDR was established in 1961 and includes information about dates and causes of death; 100% of the deaths are reported within 30 days.

Patients in the cohorts, who had symptomatic diverticular disease (DD) and at least one admission in hospital during the study period from September 15, 1997 to December 31, 2005 for women and from January 1, 1998 to December 31, 2005 for men, were compared with healthy controls in the cohort. Only incident cases were included and patients were censored after diagnosis. Outcome variables were defined in accordance with the WHO International Classifications of Diseases (ICD-10): diverticular disease was defined by a primary diagnosis of K572-9.

#### Statistical analysis and confounders

Multivariable cox regression was used to investigate the association of dietary intake of different types of dietary fibres with the incidence of hospitalisation due to DD. Each man and woman contributed details of follow-up time from entry into the cohort to the date of a diverticular disease diagnosis, or date of death from any cause or December 31, 2005, whichever occurred first. The proportional hazards assumptions were checked (by Kaplan–Meier curves) and satisfied, and Cox proportional hazards regression was used to estimate relative risks (RR) with 95% confidence intervals (CI) using the PHREG procedure in SAS (version 9.1; SAS institute, Inc., Cary, North Carolina, USA [25]). Multivariable analyses were adjusted for age (5-year age groups), diabetes (yes/no), hypertension (yes/no), BMI (< 20, 20–25, > 25 kg/m^2^), physical activity (h/day), alcohol (g/day), use of steroids (ever/never), smoking (ever/never) and educational level. We tested for linear trend across categorical models by modelling the median of each fibre quartile as a semi continuous variable and including this variable in a multivariable model.

All *p* values shown are two-sided. *p* < 0.05 was considered statistically significant for all analyses. Regarding the factors adjusted for in the multivariable model, a missing value for each specific variable was used so that the individual would not be excluded from the analyses. Data are presented separately for men and women, since they were recruited from different cohorts. All authors had access to the study data and have reviewed and approved the final manuscript.

### Ethical approval

Ethical approval for this study was given by the local ethical committee of the Karolinska Institutet (2006/147-32). The study is reported according to the criteria set out in the Strengthening the Reporting of Observational studies in Epidemiology (STROBE) [26].

## Results

During 337,919 person-years of follow up, 255 (0.57%) men in the COSM cohort were diagnosed with DD, and correspondingly 505 (1.14%) women in the SMC cohort during 287,789 person-years of follow-up. Both groups were similar in most aspects except that men had a higher proportion of diabetes mellitus and consumed more alcohol than women (Tables 1, 2). Fibre intake in quartiles was examined in relation to potential confounders and is presented in Tables 1 and 2. Smoking was more common in patients with lower fibre intake in both cohorts. Among men, in the COSM cohort, alcohol consumption was higher in patients with the lowest fibre intake. Both the cohorts were further analysed separately and divided into quartiles according to the intake of dietary fibre, intake of fibre from fruits/vegetables and from cereals separately.

### Overall intake of fibre

Women who reported the highest total dietary intake of fibres had, in multivariable analysis, a 25% decreased risk (RR 0.75, 95% CI 0.57–0.99; *p* for trend = 0.002) of hospitalisation for DD compared to those who reported the lowest amount of overall intake of fibres (Table 3).

**Table 3:** Age-adjusted and multivariable relative risks for symptomatic diverticular disease in the Swedish Mammography Cohort, 1997–2005, and the Cohort of Swedish Men, 1998–2005, by overall intake of fibres in quartiles, with Q4 representing the highest intake

	Women (*n* = 36,110)			Men (*n* = 44,723)		
	Total fibre intake, median, g	Age adj.	Multivariate^a^	Total fibre intake, median, g	Age adj.	Multivariate^a^
Q1	16.15	1 (reference)	1	21.51	1	1
Q2	19.94	0.91 (0.70–1.18)	0.93 (0.71–1.20)	27.54	0.73 (0.52–1.01)	0.76 (0.54–1.05)
Q3	23.1	0.75 (0.57–0.98)	0.77 (0.58–1.01)	32.38	0.74 (0.53–1.03)	0.79 (0.56–1.10)
Q4	28.29	0.74 (0.56–0.97)	0.75 (0.57–0.99)	39.63	0.57 (0.40–0.81)	0.61 (0.43–0.88)
*p* value	–	0.01	0.02	–	0.002	0.01

Values in parenthesis are 95% confidence intervals

^a^Cox proportional hazards regression analysis adjusted for age, smoking, BMI, education, alcohol consumption, physical activity, hypertension, diabetes mellitus and steroid use

Among men with the highest overall fibre intake, a 39% (RR 0.61, 95% CI 0.43–0.88; *p* for trend = 0.01) decreased risk of disease was seen in multivariate analysis compared to those who reported the lowest amount of overall intake of total fibres (Table 3).

### Fruits and vegetables

Women with the highest quartile intake of fruit and vegetable fibre (median 12.6 g) had a 30% decreased risk (RR 0.70, 95% CI 0.53–0.92; *p* for trend = 0.004) of disease compared to those who reported the lowest amount of intake of fruit and vegetable fibres (median 4.1 g) (Table 4). Among men, a similar trend was observed. Among those who reported the highest intake (10.3 g), a 33% decreased risk (RR 0.67, 95% CI 0.46–0.98; *p* for trend = 0.03) (Q4) was found compared to those who reported the lowest intake of fruit fibres (2.9 g) (Q1) (Table 4).

**Table 4:** Age-adjusted and multivariable relative risks for symptomatic diverticular disease in the Swedish Mammography Cohort, 1997–2005, and the Cohort of Swedish Men, 1998–2005, by intake of fruit and vegetable fibres in quartiles, with Q4 representing the highest intake

	Women (*n* = 36,110)			Men (*n* = 44,723)		
	Fruit and veg fibre median, g	Age adj.	Multivariate^a^	Fruit and veg fibre, median, g	Age adj.	Multivariate^a^
Q1	4.1	1 (reference)	1	2.85	1	1
Q2	6.46	0.87 (0.66–1.14)	0.88 (0.68–1.14)	4.84	0.93 (0.67–1.29)	0.96 (0.69–1.34)
Q3	8.74	0.68 (0.52–0.89)	0.68 (0.52–0.90)	6.82	0.86 (0.61–1.20)	0.90 (0.64–1.26)
Q4	12.56	0.70 (0.54–0.93)	0.70 (0.53–0.92)	10.25	0.65 (0.45–0.93)	0.67 (0.46–0.98)
*p* value		0.004	0.004		0.01	0.03

Values in parenthesis are 95% confidence intervals

^a^Cox proportional hazards regression analysis adjusted for age, smoking, BMI, education, alcohol consumption, physical activity, hypertension, diabetes mellitus and steroid use

### Cereals

A high intake of fibre from cereals among both women and men was not associated with the risk of hospitalisation for DD [women 0.90 (0.68–1.19); *p* = 0.5 and men 0.76 (0.53–1.10); *p* = 0.05] (Table 5).

**Table 5:** Age-adjusted and multivariable relative risks for symptomatic diverticular disease in the Swedish Mammography Cohort, 1997–2005, and the Cohort of Swedish Men, 1998–2005, by intake of cereal fibres in quartiles, with Q4 representing the highest intake

	Women (*n* = 36,110)			Men (*n* = 44,723)		
	Cereal fibre, median, g	Age adj.	Multivariate^a^	Cereal fibre, median, g	Age adj.	Multivariate^a^
Q1	7.44	1 (reference)	1	12.83	1	1
Q2	10.1	0.87 (0.66–1.14)	0.89 (0.67–1.17)	17.81	1.02 (0.74–1.41)	1.07 (0.77–1.47)
Q3	12.35	0.89 (0.68–1.17)	0.92 (0.70–1.22)	22.12	0.69 (0.49–0.99)	0.74 (0.52–1.06)
Q4	15.95	0.90 (0.69–1.18)	0.90 (0.68–1.19)	28.82	0.72 (0.50–1.02)	0.76 (0.53–1.10)
*p* value		0.5	0.5		0.01	0.05

Values in parenthesis are 95% confidence intervals

^a^Cox proportional hazards regression analysis adjusted for age, smoking, BMI, education, alcohol consumption, physical activity, hypertension, diabetes mellitus and steroid use

## Discussion

This major prospective cohort study among middle-aged men and women demonstrates that high intake of dietary fibre, especially from fruits and vegetables, reduces the risk of hospitalisation for diverticular disease.

In general, fruits and vegetables contain higher level of cellulose than cereals. As an insoluble fibre, cellulose represents an average of 30% in fruits and 50% in vegetables. Insoluble fibres are metabolised by colonic bacteria less than (water-) soluble fibres. Insoluble fibres can increase faecal output by acting as a fermenter, by stimulating microbial growth resulting in the production of short chain fatty acids (SCFA). SCFA are known as an important fuel source for the colon in general and particularly in sigmoid colon [27]. This might partially explain the observed beneficial effect of fibre from fruits and vegetables in our study.

According to the results in the present study, the difference between women eating the highest and lowest amount of fibres from fruit and vegetables, was 8.5 g/day (12.6 − 4.1 g/day). This is how much extra fibre one must eat every day to achieve a risk reduction of 30%. For comparison, an ordinary sized apple or orange contains 3–3.5 g of fibre. For men, the corresponding difference was 7.4 g/day to achieve a risk reduction of 35%.

When discussing the role of fibres in preventing diverticular disease, it is important to define where in the progress of the disease an increased fibre intake might be beneficial. Risk factors for developing asymptomatic findings of diverticulosis might differ from risk factors affecting symptomatic disease. Firstly, Painter and Burkitt [8] and later others [28] found that a low fibre intake could promote the formation of colonic diverticulosis when comparing high fibre-consuming native Africans with populations in the Western world with a low fibre intake.

In contrast, Peery et al. [9] recently postulated that a high fibre diet could promote diverticulosis in a study of 2104 patients undergoing an extensive diet interview using validated food frequency questionnaires, 3 months after a colonoscopy-proven diagnosis of asymptomatic diverticulosis had been established and communicated to the patients.

Secondly, it has been shown that a high fibre intake could prevent symptomatic diverticular disease, mainly diverticulitis. Aldoori et al. found that the insoluble component of dietary fibre was inversely associated with risk of diverticular disease among male health professionals in the USA [6]. This finding is contradictory to the present study in which fibres from fruits and vegetables were shown to be most beneficial. Yet, the outcome of the study of Aldoori differed because the present study included only hospitalisations for DD, compared to self-reported symptomatic DD in the study of Aldoori et al. This might partly explain the diverging results.

The results in the present study confirm the recently reported findings by Crowe et al. [17], though they also reported that fruit could be a more beneficial fibre contributor compared to vegetables. Our data did not allow us to examine the effect of fruits and vegetables separately.

Thirdly, studies have also addressed the potential benefit from high fibre intake to prevent recurrences after an episode of medically managed diverticulitis. Brodribb et al. [29] found a reduction of symptoms after 3 months with fibre supplement in a placebo-controlled trial. However, other studies have been contradictory [9].

Other significant risk factors for symptomatic DD in the diet, high intake of red meat and fat, have been postulated, though the relationships are weak [11]. Also, previous data have stated smoking, low physical activity and obesity to increase the risk for DD [12, 14]. Nuts, seeds and corn do not seem to affect the risk [30]. Recently, two studies of mono- and dizygotic twins have found a hereditary component in the development of DD, with a genetic effect up to 40% [3, 4]. Medications might also affect the incidence of DD. Use of corticosteroids, both inhaled and orally, increase the risk of hospital admission because of DD, according to a recent report, and we therefore included use in the multivariate analysis [13]. Yet, the effect of non-steroid anti-inflammatory drugs (NSAIDs) and acetylsalicylic acid (ASA) is contradictory. A previous study in the SMC cohort could not explore any effect of these medications on the incidence of DD and was therefore omitted in our analysis [13].

The strengths of the present study are the large numbers in the cohorts, independent prospective data ascertainment, homogenous classification of exposures and outcomes. Potential confounders such as smoking, BMI, physical activity, use of steroid medication and co-morbidity were included in the multivariate analysis and almost complete follow-up was achieved through national registers.

There are limitations of our study, since there is information bias in our questionnaires and low response rate of answering among the men in the COSM cohort. Diet intake was assessed only at baseline and may not reflect the actual intake during the whole study period. Potential confounders such as smoking, BMI, educational level and physical activity were included in the multivariate analysis, but there is a possibility that unmeasured or residual confounders may have biased our findings. Also, disease classification could have been flawed. The final diagnosis was made by a clinician and stated in the discharge summary, and then subsequently reported to the National Patient Register. In the present study, men and women with mild symptoms were likely missed, [13], as only those with symptomatic disease requiring in-hospital care were included. Diverticular bleeding has no separate code in ICD 10 and such events are therefore most likely missed. Moreover, fluid intake might be a potential confounder. Unfortunately, our collected data regarding fluid intake were not complete. Water was available as a calculated variable from all dietary questions in the survey. However, there were no data of how much water they drank. Therefore, we could not control for this in the analysis.

For validation purposes, 528 consecutive patients discharged (at least once) from Danderyd Hospital in Stockholm, Sweden, with a primary discharge code of K572-9 were re-evaluated [12]. In all, 95.8% were correctly classified as having symptomatic diverticular disease and misclassification was found in 4.2% only. This is in line with a previous Swedish report [31].

To conclude, high intake of fruits and vegetables may reduce the risk of hospitalisation due to DD in this major cohort study of both men and women, while intake of cereals does not influence the risk.

## Abbreviations

DD: Diverticular disease
SMC: Swedish Mammography Cohort
COSM: Cohort of Swedish Men
